# A novel development to encrypt data communication under t-intuitionistic fuzzy environment

**DOI:** 10.1371/journal.pone.0308140

**Published:** 2024-09-27

**Authors:** Hanan Alolaiyan, Laila Latif, Umer Shuaib, Abdul Razaq, Qin Xin

**Affiliations:** 1 Department of Mathematics, College of Science, King Saud University, Riyadh, Saudi Arabia; 2 Department of Mathematics, Government College University, Faisalabad, Pakistan; 3 Department of Mathematics, Division of Science and Technology, University of Education, Lahore, Pakistan; 4 Faculty of Science and Technology, University of the Faroe Islands, Torshavn, Faroe Islands, Denmark; University of the West of Scotland, UNITED KINGDOM OF GREAT BRITAIN AND NORTHERN IRELAND

## Abstract

The field of cryptography has grown significantly with the advent of information and communication technologies due to the increasing complexity of cyber threats and rising security requirements. This evolution has come with the creation of new cryptosystems and improvements to current ones. This study is the first to explore the RSA approach in the framework of t-intuitionistic fuzzy subgroups. This technique makes group-based cryptographic operations safer when there are unclear relationships and hesitations. This supports the complex and uncertain nature of subgroup membership, allowing for much more significant representations of the degrees of belonging, non-belonging, and hesitancy for the group elements along parameter ’t’. The t-intuitionistic fuzzy RSA technique employs a t-intuitionistic fuzzy subgroup to address cryptosystem ambiguity, fuzziness, and imprecision. Consequently, inaccurate cryptographic data is more effectively represented, manipulated, and protected. Furthermore, this technique enhances the current level of fuzzy cryptography. The t-intuitionistic fuzzy RSA algorithms are of theoretical and practical value, as they significantly contribute towards developing fuzzy cryptography, fuzzy algebraic structures, and decision support systems. In this paper, the notions of t-intuitionistic fuzzy numbers and triangular t-intuitionistic fuzzy numbers are introduced. A new RSA cryptosystem based on a t-intuitionistic fuzzy subgroup is proposed in which the plaintext and the ciphertext are obtained in terms of t-intuitionistic fuzzy numbers and triangular t-intuitionistic fuzzy numbers. In addition, the significance of the concept of the t-intuitionistic fuzzy subgroup is highlighted as a suitable alternative tool to secure the data under consideration. In addition, the practical effect of the proposed methods is also investigated in this study. A mathematical mechanism is presented to implement the t-intuitionistic fuzzy RSA algorithm. Finally, a comparative analysis of the developed technique is presented with some existing methods to showcase the applicability and superiority of the recently developed method.

## 1. Introduction

### 1.1. Background

Today’s digital world requires cryptography to protect data. It protects communication channels, prevents unauthorized access to critical data, and verifies user identities and transactions [1–3]. The study of cryptography allows senders and recipients to communicate secure and secret messages. The data is encrypted with a private key and coded before being sent to the sender for decryption. This terminology needs data security and policies to prevent unauthorized access to and reading private correspondence. Data confidentiality, integrity, user authentication, and non-repudiation need cryptography. This technology is essential for financial transactions and data security [4,5]. Public key encryption secures account numbers, transaction volumes, digital signatures, and credit card authorizations in electronic transactions. The technology secures websites and electronic communications. For a website to be secure, it must exchange and save every piece of data transmitted between computers in encrypted form, and our electronic transactions are provably trustworthy. The advent of digital communications makes digitally signed and digitally notarized communications possible, thus drastically enhancing the dependability of our electronic transactions in proving the origin and authenticating the integrity of our digital communications. Cryptography is needed to protect privacy and data integrity. For authentication and non-reliability of digital transactions, it is necessary to establish the trust upon which our digitally interconnected and digitally interdependent world depends in an age where digital communications and networked connectivity are everywhere [6,7]. Numerous researchers have devised novel approaches regarding this terminology. A framework for an artificial neural network to generate unique ciphers was presented by Blackledge et al. [8]. Their study demonstrated that neural networks are practical cryptographic tools that can be used to develop personalized ciphers that satisfy the necessary security requirements. In addition, Chakraborty et al. [9] developed a comprehensive portrayal of the principles and applications associated with network security. The RSA algorithm is a widely used asymmetric cryptographic technique introduced by its creators, Rivest, Shamir, and Adleman [10]. Cryptosystems based on the RSA algorithm are used for secure connections and digital signatures. The RSA algorithm is still the most robust encryption method widely used online. This algorithm is based on the mathematical properties of large prime numbers. Due to the number of distinct systems that attach to the RSA system to maintain its structure and preserve the confidentiality and integrity of digital data, many practical applications and modifications of its basic algorithms have been proposed recently. Therefore, it has become one of the most essential parts of cryptography because of its innovative approach to establishing secure communication using public-key mechanisms. The algorithm encrypts data transmission across an untrusted network when used with a shared secret key without requiring a secret key first to be shared. By enabling a secure, shared key exchange, the system overcomes security issues by trying to distribute keys and provides for authentication and digital signatures. This algorithm also formed the hybrid cryptosystem, allowing the security industry to benefit from giving hundreds of thousands of users secure data transmission, integrity verification of transmitted messages, digital signature generation and verification systems, and the exchange of shared keys to achieve privacy for communication and authentication. The RSA encryption method is secure due to the use of prime numbers. The RSA algorithm allows two parties to exchange messages privately. It is based on factors in large numbers and among secure encryption methods generally used in secure email, digital signatures, and online banking. Over the years, mathematicians have developed many additional methods to improve the basic RSA algorithm. Chavan et al. [11] discussed a method for data security authentication in a network using a combination of symmetric and asymmetric algorithms. Blackledge and Mosola [12] provided a brief explanation of artificial intelligence, which is used for the encoding and cryptanalysis of encrypted data to evaluate the robustness of a cryptographic technique. Mathematicians have created RSA or hybrid cryptosystems in the past, using a variety of methods to secure and implement the system, including the use of short secret key exponents [13], the application of the Chinese Remainder Theorem (CRT) during the decryption process [14], the inclusion of homomorphism properties [15], the use of four large primes [16], the use of double additive string rates [17], and the implementation of n primes and bit padding [18]. Cryptosystems in the fuzzy framework are designed for uncertainty and imprecision, which naturally exist in real-life data. Under this condition, typical cryptographic methods fall short, based on "either/or" or "how much" information, which readily degrades to an impractical form. Fuzzy logic allows cryptosystems the flexibility to represent uncertainty and adapt suitably a cryptosystem to the differing levels of ambiguity and model better human perception. Uncertain contexts have been added to address the lack of clarity around cryptosystem approaches in the academic literature. While the literature discusses various techniques for uncertain data, each approach has strengths and weaknesses.

### 1.2. Literature review

Practical situations often raise challenges that cannot be easily addressed with binary logic. Consider today’s automated vehicle speed control systems; accurately differentiating between ‘slow,’ ‘moderate,’ and ‘fast’ velocities can be arduous. To solve this kind of issue, Zadeh [19] introduced the concept of fuzzy sets (FSs) arising from the complexity and uncertainties inherent in real-world scenarios. This inadequacy of binary sets in such circumstances has made clear the significance of FSs. FSs allows the representation of the membership grades for elements that do not necessarily belong to the set, a beneficial capability for managing ambiguity and irregularity in scenarios such as those described. The FSs has become a widely applied framework for representing the cumbersome variables of traditional crisp binary structures in fields like artificial intelligence and control systems. Computational cognition was thus generated from interpretative information, and computational perception was assumed to have intrinsic ambiguity, inherent imprecision, non-exactly bounded, and partially inaccurate information. It has been implemented in various disciplines as a general framework for describing variables to deal with variables difficult to categorize using traditional binary sets. This is the base of information available in computational cognition and perception. In this knowledge-based system, a system with a fully equipped suite of sensors can make its way deftly through confusing differentiation and regulate its speed as a function of lane information, spacing and density, and road surface fairness. This knowledge can [20–22] be used advantageously in many diverse disciplines, such as industrial automation, image processing, control system engineering, decision-making, robotics, power engineering, optimization, and consumer electronics. When uncertainty or ambiguity pertains to quantitative values instead of qualitative distinctions in isolation, fuzzy numbers (Fℕs) become an essential requirement. Fℕs incorporates quantities in the form of numbers into the concept of FS, which is primarily concerned with degrees of membership. FNs were initially developed by Zadeh [23,24]. As a consequence, a more accurate representation is achieved across multiple domains. Fℕs, for instance, enables a more complex quantification in the domain of financial risk assessment, where the degree of risk cannot be simplistically categorized as low or high. Fℕs is highly advantageous in temperature control systems that utilize precise temperature ranges to distinguish between "warm" and "hot" temperatures. Fℕs are significant in FS theory as they provide a generalized platform for expressing imprecise, incomplete, and inconsistent information. Fuzzy logic is rendered more practical and precise by incorporating Fℕs when quantitative imprecision is crucial for analysis and decision-making. In 2022, Betta [25] proposed a customer requirements management model using Fℕs. It provides the best management strategies resulting from carefully interpreting fuzzy parameter estimates. In applications, the most commonly used Fℕs are the triangular and trapezoidal shapes, defined by Buckley and Eslami in [26]. Fuzzy triangular, trapezoidal, and their generalized form are a few common notions by which the linguistic variables are quantified. Mukherjee et al. [27] conducted a comprehensive examination of the arithmetic operations of FNs using the alpha-cut method in a novel approach and provided their interpretation of the results. Kahraman [28] discussed the application of FS theory in industrial engineering, highlighting new tools and ideas developed by researchers to address problems specific to industrial engineering. However, the FS theory, characterized by only one function, the "membership function," in most cases cannot be used fully to express some complex fuzzy information. For example, during voting, if ten people are voting for an issue, three of them give the ‘agree,’ four of them give the ‘disagree,’ and the others abstain. FS cannot fully express the polling information. To solve this problem, Atanassov [29] developed the concept of intuitionistic fuzzy sets (IFSs), a generalization of FS. The theory of IFS differs from FS in that they include a membership degree, non-membership degree, hesitancy degree, or intuitionistic index. These aspects align with humans who express decisions using negation, affirmation, or hesitation. Compared to uncertainty theories that primarily focus on quantitative problem-solving, the utilization of IFS offers distinct advantages. This approach presents a flexible framework that effectively handles decision-making processes’ inherent ambiguity and uncertainty. The IFS theory is handy in various fields, such as decision-making problems [30–36], logic programming [37,38], medical diagnosis [39–43], and career determination [44–47]. Kozae1 et al. [48] reviewed the definitions of IFS and suggested its implementation in COVID-19. Citil [49] proposed the application of an IFS in determining high school using distance measures. Jars et al. [50] reviewed the IFS concept and presented a useful selection method in various disciplines. Aggarwal et al. [51] studied the significance of this idea in making housing purchase decisions in society. In addition, Ejegwa et al. [52] gave a comprehensive note on some selected models of IFS in real-life situations, such as pattern recognition, carrier determination, and diagnostic medicine. Gu and Zhang [53] proposed a new method for project risk assessment based on this knowledge. The multi-attribute decision problem in an intuitionistic fuzzy environment was discussed by Junhang et al. [54]. A text classification framework based on IFS similarity measures was presented by Intrarapaibon [55]. The need for intuitionistic fuzzy numbers (IFℕs) instead of the traditional Fℕs arises from the requirement for a deeper and richer explanation of the uncertainties enveloping a particular situation. Unlike Fℕs, which represent merely membership degrees, IFℕs also account for non-membership degrees and degrees of hesitancy, which makes them invaluable in circumstances closer to reality, where uncertainties are multiple and have several dimensions. For example, in the estimation of the completion time of a project, an IFℕ may mean a relatively good chance of finishing in time according to some expert estimation, be well aware of a few possible delays but still exhibit a level of hesitancy concerning the estimation. This richer representation helps us understand the chance of some event, the likely deviations, and our confidence in it. Hence, it makes IFℕs more appropriate for complex decision-making contexts where uncertainties are all-pervasive, multi-dimensional, and multi-faceted. Triangular IFℕ is a more generalized platform for expressing imprecise, incomplete, and inconsistent information when solving multi-criteria decision-making (MCDM) problems and for expressing and reflecting the evaluation information in several dimensions. Seikh et al. [56] defined the notions of IFℕs and triangular IFℕs. They also presented the decision-making problem based on these concepts. In modelling the decision-making problem with imprecise quantity, one approach is to deal with such imprecise quantity as the IFℕ. Thus, the intuitionistic fuzzy optimization problem involves the comparison of Fℕs. The comparison of these imprecise numbers needs to develop the ranking methods for IFℕs. The ranking of IFℕs plays an important role in comparing IFℕs decision-making problems. Bharati [57] defined a new ranking function, particularly for triangular IFℕ, and has verified its axioms. Aikhuele and Odofin [58] proposed a methodology for addressing MCDM problems in which triangular IFℕs represent the performance ratings. Researchers have provided several methods for ranking IFℕs and triangular IFℕs in an uncertain environment for solving MCDM problems [59–62]. The authors have also compared their approach with some existing methods to demonstrate its suitability in the face of uncertainty. There are many situations when conventional IFℕs might not accurately reflect the complexities under consideration. For example, suppose we are interested in approximating the time required to complete a given project. An IFℕ may indicate a relatively heightened likelihood of finishing the job on time, but with an understanding that certain obstacles could pop up unexpectedly or that some degree of uncertainty or resistance is present in the estimate. IFℕs might not accurately represent this emphasis. When the dichotomy is the completion of a project six or seven days from now, for example, where timeliness is obviously of the essence, these are the sorts of concerns a decision-maker should consider. To address concerns of this sort, we introduce the notion of t-intuitionistic fuzzy number (t-IFℕ) in this paper. By accounting for the parameters’ t,’ decision-makers can adjust the interrelationships between membership, non-membership, and hesitancy to reflect the particular requirements of the domain or venture under consideration. As such, the modelling approach is more accurate and adaptable for use in intricate decision-making situations in which specific operational or contextual details influence the interconnections among these three components. In certain situations, IFS may not be capable of managing intricate decision-making processes. For example, consider the selection committee tasked with appointing a faculty member for a university. Some committee members may declare that an applicant is suitable for the position if their research work is at least 90% superior to the other applicants. However, other committee members may approve applicants for the position if they perform at least 80% better than their peers during the interview. To handle these situations, Sharma [63] introduced the theory of the t-intuitionistic fuzzy sets (t-IFS). The theory of the t-IFS acts as a pivotal approach to handling the imprecision in the data by adding a parameterizing factor during the process, as compared to the theory of IFSs. By incorporating satisfaction levels (acceptance), rejection, abstention, and priorities (parameter "t") linked to objectives, this methodology simplifies the decision-making process, improves the coherence of reasoning, and reduces subjectivity. The adjustability of the parameter t’ provides flexibility that enables decision-makers to emphasize particular aspects of uncertainty to a greater or lesser degree depending on the demands of a particular situation. The t-IFSs inherent flexibility makes them an indispensable tool for handling complex relationships and varying degrees of uncertainty. The t-IFSs are a more explicit and context-dependent representation than conventional IFSs. The framework is beneficial because it relates to decision-making involving vagueness and uncertainty. An extensive study of the t-intuitionistic fuzzy subgroup (t-IFSG) may be found in [64,65].

### 1.3. Research gap

Traditional encryption approaches may struggle when dealing with imprecise, confusing, or ambiguous data. The goal of classical cryptography is to convert data into an accurate, binary distinction that may not match real-world data with variable degrees of uncertainty. Many academics have created fuzzy formulations to solve these algorithms. Mandanyake et al. [66] created a fuzzy logic-based encryption technique that may be low-processing or high-security, depending on user needs. Abdullah et al. [67] designed the RSA cryptosystem for FS. Many mathematicians have developed fuzzy cryptosystem data security techniques. These techniques were explored in [68–70]. However, Shor’s quantum computing assaults, which process massive targets, may restrict the RSA method, which secures communications. Power consumption or cryptographic timing might be used to conduct RSA side-channel attacks. As these key numbers expand, computing efficiency and resource allocation may alter, exposing scaling concerns. However, RSA’s fuzzy foundation makes it more resilient to unforeseen events. We may increase computational complexity and needs and reduce effectiveness by introducing limits detected in this process. Thus, FS may need more computing complexity and less efficacy with higher fuzziness to complete a full quantitative evaluation. In the fuzzy RSA framework, we deal only with membership degrees. The fuzzy framework in RSA may not accurately represent complex information, presenting new challenges for theoretical research and practical demonstration. The flexible RSA methodology of ambiguous intuitionistic logic better conveys uncertainty by incorporating non-membership degrees. Due to the subjective nature of IFSs, formal analysis and proof are incredibly hard. The exact impact of non-membership degrees on security and performance is currently unknown. The incorporation of an additional parameter has the potential to introduce complexity and slow computations. The RSA methodology is vulnerable to compromise by side-channel attacks and quantum computation. The RSA fuzzy framework’s computational complexity limits its ability to improve resilience. Furthermore, non-membership degrees complicate verifying and validating the RSA intuitionistic fuzzy framework. The safety and efficacy of the approach are currently outside the bounds of scientific knowledge. This assertion must be carefully considered: IF-RSA safeguards sensitive data using numerical values of 0.8, 0.15, and 0.05, representing minimal risk, practically total security, and less accurate reservations. Policymakers may modify algorithms to meet security standards or pose a risk environment. The feature might help address some of the security uncertainties and weaknesses. To cope with all these issues, we invented the t-intuitionistic fuzzy RSA (t-IF-RSA) method to deal with them. The main objective for developing t-IF-RSA is to resolve the constraints of intuitionistic fuzzy frameworks, conventional fuzzy frameworks, and classical RSA algorithms. The adaptability and effectiveness of this novel t-IF-RSA algorithm are its two principal characteristics. By adjusting the "t" parameter of the t-norm and t-conorm operators, security could be guaranteed. It is of the utmost importance to contemplate the benefits of deception in situations where the outcome of the parameter ‘t’ is heavily dependent on the accuracy of the message. While it may not provide exhaustive safeguards against every conceivable danger, it can potentially assist law enforcement agencies in their endeavors to identify and apprehend individuals who eavesdrop. Security measures intended to protect fewer sensitive data often serve as sites of vulnerability for the system. In light of these modifications, scholars have examined many potential strategies for fortifying the default RSA configuration. The result could be more readily understandable for the majority of users. In this case, the "t" parameter has an enormous benefit: it permits multiple security-level configurations and modifications to those configurations. By adjusting the parameter "t," individuals can fine-tune the time spent on their feet in cryptographic terminology during interrogations concerning cryptographic ambiguity. The authors state that this enables a comprehensive range of t-norm and t-conorm operators to model imperfections and provide considerable flexibility in contexts where a monolithic approach is unsuitable, such as managing uncertainty. The use of t-IF-RSA has greatly enhanced the work. It is necessary to evaluate how well the algorithm will perform and how it will detect errors at different levels of uncertainty. This is done by making use of the t-norm and t-conorm operators. Data safeguarding is complex; however, t-IF-RSA has an advantage over other systems. In the traditional RSA algorithms, the security levels are constant, whereas with t-IF-RSA, this is not so, and it is also fully customizable. Cryptographic professionals can change the algorithms to match their security needs and risk composition, a potent property, as is the fact that the innovative, versatile manner is initiated by the parameter "t" and recapitulates wider solutions of IFSs to augment precision. The introduction of t-IF-RSA is feasible either as an alternative cryptographic system or through any approach that evaluates aspects of a system if it employs RSA algorithms. The significance of the RSA algorithm has motivated us to integrate the t-IFS theory into the algorithm of the original RSA cryptosystem. The t-IF-RSA algorithm can be utilized in web browsers, email, virtual private networks (VPN), chat, and other communication channels. Furthermore, this newly defined algorithm can establish a secure connection between VPN clients and the VPN server. This new approach can exchange keys and establish a secure channel under protocols such as open VPN and transport layer security (TLS) handshakes. The main objective of this research is to improve the security level of data transmission with a new encryption technique based on public and private keys created by the t-IF-RSA algorithm.

### 1.4. Novelty of the current study

The novelty of the current study is explained in the subsequent discussion:

- The t-IFℕ enhances the traditional IFℕ by including the parameter "t," which enables a more accurate representation of uncertainty. This work offers the possibility of a richer action of levels of uncertainty, imprecision, and vagueness, providing decision-makers with the flexibility of a conceptual and computational tool for representing and managing complex degrees of partial ignorance. Enhanced capabilities for decision-makers to communicate their subtle judgments will, in turn, lead to the formulation of more informed and vigorous decisions.
- The newly developed triangular t-IFℕ implementation that leverages the flexibility of the t-intuitionistic fuzzy setting and the rich characteristics of triangular IFℕ provides an alternative method to offer decision-makers working under vagueness, imprecision, uncertainty, and partial truth a more pragmatic and efficient tool to allow a compelling presentation of uncertainties, thus economizing computational resources, increasing the facility with which complex judgments can be communicated, and, ultimately, allowing much more informed and robust decisions to be made. The new convergence measures can be utilized to drastically enhance optimization capabilities in many applications, including but not limited to machine learning, engineering design, quality control, decision-making, and cryptography.
- The technique of t-IF-RSA based on t-IFSG is designed to overcome the uncertainty of sub-groups, enhancing the cryptographic security. By combining the flexibility and adaptability with the security of t-IFSG is altered, which is known as parameter "t." This parameter diminishes the subgroup uncertainty, resulting in a dynamic and adaptable cryptographic solution. The unique t-IF-RSA technique of t-IFSG is employed to manage the ambiguity of subgroups. The variation in security levels is achieved using the subgroup properties concerning the included parameter ‘t’. This innovative modification significantly improves cryptosystem performance. It allows the subgroups to enhance and adapt to security while they become tenuous and complex.

### 1.5. Motivations and contributions of the current research

The subsequent discussion comprehensively explains the motivations and contributions that enhance the current study.

- The inception of the concepts of t-IFℕ and triangular t-IFℕ. The strength of these notions is that they can encapsulate ambiguity, uncertainty, and imprecision in thought and decision-making. They provide an organized and transparent framework to model complex real-world scenarios that surpass the capabilities of traditional modeling tools. The concepts will benefit engineering design, quality control, decision-making, finance, risk assessment, and cryptography. The intrinsic t-IFℕ supports uncertainty modeling for exploiting uncertainty in complex real-world applications, integrating expert knowledge, and managing ambiguous data. Increasing the complexity associated with these notions of encryption and decryption makes it difficult for potential attackers to estimate or manipulate signals. Compared with conventional statistics, these notions emulate natural unpredictability and uncertainty from different sources and their usual accurate interpolation. A t-IFℕ with these properties may not allow an attacker or an eavesdropper to sense and monitor trends and could be robust in identifying the vulnerability of the communications.
- Development of the t-IF-RSA algorithm in the framework of t-IFSG. In software applications where the data is ambiguous or uncertain, integration of the RSA algorithm with the t-IFSG architecture will enhance security and privacy in the system. So, it is necessary to implement t-IFSG in secure communication, access control, and data protection. In the case of emerging intelligent contracts and blockchain applications, where smart contracts execute and sensitive data is encrypted, integrating both technologies in the applications using t-IFSG is a requirement, as is the case with RSA encryption. The t-IF-RSA method introduced in this paper is vital among subgroups, providing an adaptable and configurable cryptosystem solution. The introduction of parameter "t" in the IFS paradigm framework equips it with more adaptability against unclarity; hence, adaptive changes can be made upon subgroup descriptions. This technology seems to be a significant segue to cryptosystem solutions in cryptographic contexts with ambiguous subgroups and for enhanced security protocols.
- For cryptographic purposes, the t-IF-RSA algorithm is implemented in C++. Implementing the t-IF-RSA algorithm in C++ yields an individualized and flexible cryptographic solution. Its significance is that it becomes possible to selectively manipulate the utilized parameters, particularly the "t" parameter, in the face of various uncertainties. This implementation provides an adaptable security strategy that permits the solution to be applicable in a wide range of cryptographic contexts by simply enhancing resilience. C++ allows a security protocol to be implemented efficiently with robust execution. It enables a resilient security protocol to be seamlessly integrated. As a result, the t-IF-RSA algorithm is an effective safeguard mechanism for sensitive data across a diverse range of application areas.

### 1.6. Structure of the paper

The structure of the paper is organized as follows: The second section contains basic definitions that are essential for understanding the main results of this article. In Section 3, we define the notions of the t-IFℕ and the triangular t-IFℕ. In Section 4, we propose a t-IF-RSA algorithm and present an example of this proposed algorithm. In Section 5, we implement the t-IF-RSA algorithm computationally. Moreover, we establish a comparative analysis of newly proposed and existing algorithms in Section 6. This section also contains the limitations of the proposed algorithm. Finally, the conclusion and future work is described in Section 7.

## 2. Preliminaries

This section contains a brief review of some basic notions, which play a fundamental role in understanding the novelty of this article.

**Definition 1** [29]. An intuitionistic fuzzy set (IFS) *A* of a universe U is a triplet of the form: $A=\left\{\left(a_{1},\;\mu_{A}\left(a_{1}\right),\;\nu_{A}\left(a_{1}\right)\right):a_{1}\in U\right\},\;$ where *μ*_*A*_ and *ν*_*A*_ are the membership and non-membership functions defined from the universe U to [0, 1], respectively, that satisfy the condition: 0 ≤ *μ*_*A*_ (*a*_1_) + *ν*_*A*_ (*a*_1_) ≤ 1.

**Definition 2** [29]. The (*α*, *β*)–cut set of an IFS *A* is defined as follows:

$$C_{\left(\alpha ,\beta \right)}\left(A\right)=\left\{a_{1}\in U:\mu_{A}\left(a_{1}\right)\geq \alpha \wedge \nu_{A}\left(a_{1}\right)\leq \beta \right\},$$

where *α*, *β* ∈ [0, 1] and 0 ≤ *α* + *β* ≤ 1.

**Definition 3** [31]. An IFS A is an intuitionistic fuzzy number (IFℕ) of the set of real numbers ℝ, that satisfies the following properties:

1. A is an intuitionistic fuzzy normal set, i.e., *μ*_*A*_ (*a*_1_) = 1 and $\nu_{A}\left(a_{1}\right)=0,a_{1}\in R.$
2. Each (*α*, *β*)–cut set is a closed interval for every *α*, *β* ∈ (0,1].
3. The set $A=\left\{a_{1}\in U:\mu_{A}\left(a_{1}\right)>0\wedge \nu_{A}\left(a_{1}\right)<1\right\}$ is bounded.

**Definition 4** [31,56]. A LR-type IFℕ ***A*** is characterized by its membership and non-membership functions, which can be defined in the following way:

$$\mu_{A}\left(z_{1}\right)=\left\{\begin{array}{c} \begin{array}{ccc} 0 & if & z_{1}\in \left(-\infty ,m_{1}-\alpha_{1}\right]\\ L\left(\frac{m_{1}-z_{1}}{\alpha_{1}}\right) & if & z_{1}\in \;\text{(}\;m_{1}-\alpha_{1},m_{1}),\;\alpha_{1}>0\\ 1 & if & z_{1}=m_{1} \end{array}\\ \begin{array}{ccc} R\left(\frac{z_{1}-m_{1}}{\beta_{1}}\right) & if & z_{1}\in (\;m_{1},m_{1}+\beta_{1}),\;\beta_{1}>0\\ 0 & if & z_{1}\in \left[m_{1}+\beta_{1},\infty \right) \end{array} \end{array}\right.,$$

and

$$\nu_{A}\left(z_{1}\right)=\left\{\begin{array}{c} \begin{array}{ccc} 1 & if & z_{1}\in \left(-\infty ,m_{1}-\alpha_{1}^{\prime }\;\right]\\ 1-L\left(\frac{m_{1}-z_{1}}{\alpha_{1}^{\prime }\;}\right) & if & z_{1}\;\in \;\text{(}m_{1}-\alpha_{1}^{\prime },\;m_{1}),\;\alpha_{1}^{\prime }\;>0\\ 0 & if & z_{1}=m_{1} \end{array}\\ \begin{array}{ccc} 1-R\left(\frac{z_{1}-m_{1}}{\beta_{1}^{\prime }\;}\right) & if & z_{1}\in \left(m_{1},m_{1}+\beta_{1}^{\prime }\right),\beta_{1}^{\prime }>0\\ 1 & if & z_{1}\in \left[m_{1}+\beta_{1}^{\prime },\infty \right) \end{array} \end{array}.\right.$$

Note that *L*(1) = *R*(1) = 0 and *L*(0) = *R*(0) = 1. The IFℕ A is represented as follows: $A_{IFN}=\left(m_{1},\alpha_{1},\beta_{1};\alpha_{1}^{\prime },\beta_{1}^{\prime }\right)$.

**Definition 5** [31,56]. A triangular IFℕ that possesses membership and non-membership functions meets the condition of 0 ≤ *μ*_*A*_ (*z*_1_) + *ν*_*A*_ (*z*_1_) ≤ 1. These functions can be presented in the following manner:

$$\mu_{A}\left(z_{1}\right)=\left\{\begin{array}{c} \begin{array}{ccc} 0 & if & z_{1}\in \left(-\infty ,m_{1}-\alpha_{1}\right]\\ 1-\left(\frac{m_{1}-z_{1}}{\alpha_{1}}\right) & if & z_{1}\in \;\text{(}\;m_{1}-\alpha_{1},m_{1}),\;\alpha_{1}>0\\ 1 & if & z_{1}=m_{1} \end{array}\\ \begin{array}{ccc} 1-\left(\frac{z_{1}-m_{1}}{\beta_{1}}\right) & if & z_{1}\in (\;m_{1},m_{1}+\beta_{1}),\;\beta_{1}>0\\ 0 & if & z_{1}\in \left[m_{1}+\beta_{1},\infty \right) \end{array} \end{array}\right.,$$

and

$$\nu_{A}\left(z_{1}\right)=\left\{\begin{array}{c} \begin{array}{ccc} 1 & if & z_{1}\in \left(-\infty ,m_{1}-\alpha_{1}^{\prime }\;\right]\\ \frac{m_{1}-z_{1}}{\alpha_{1}^{\prime }\;} & if & z_{1}\;\in (m_{1}-\alpha_{1}^{\prime },\;m_{1}),\;\alpha_{1}^{\prime }\;>0\\ 0 & if & z_{1}=m_{1} \end{array}\\ \begin{array}{ccc} \frac{z_{1}-m_{1}}{\beta_{1}^{\prime }\;} & if & z_{1}\in \left(m_{1},m_{1}+\beta_{1}^{\prime }\right),\beta_{1}^{\prime }>0\\ 1 & if & z_{1}\in \left[m_{1}+\beta_{1}^{\prime },\infty \right) \end{array} \end{array}\right..$$

where $\alpha_{1}^{\prime }\leq \alpha_{1}\leq m_{1}\leq \beta_{1}\leq \beta_{1}^{\prime }$. Moreover, *m*_1_ denotes the mean value of *A*. The triangular IFℕ is symbolized as follows: $A_{IFN}=\{m_{1},\alpha_{1},\beta_{1};\alpha_{1}^{\prime },\beta_{1}^{\prime }\}.$

**Definition 6** [56]. Let *A* be a triangular IFℕ. The (*α*, *β*)–cut interval of *A* is defined as:

$$C_{\left(\alpha ,\beta \right)}\left(A\right)=\left\{\left[\left(m_{1}-\alpha_{1}\right)+{\alpha \alpha }_{1},\left(m_{1}+\beta_{1}\right)-\alpha \beta_{1}\right];\left(\left.-\infty ,m_{1}-\alpha_{1}^{\prime }\beta \right]⋃\left[\left.m_{1}+\beta_{1}^{\prime }\beta ,\infty \right)\right.\right.\right\}.$$

where *α*, *β* ∈ [0,1] and 0 ≤ *α* + *β* ≤ 1.

**Definition 7** [63]. For any IFS *A* of a universe U and 0 ≤ *t* ≤ 1, we say that *A*_*t*_ is t-intuitionistic fuzzy set (t-IFS) of U with respect to *A* and is defined as:

$$\mu_{A_{t}}\left(a_{1}\right)=\text{min}\left\{\mu_{A}\left(a_{1}\right),t\right\}\;\text{and}\;\nu_{A_{t}}\left(a_{1}\right)=\text{max}\left\{\nu_{A}\left(a_{1}\right),\;1\text{-}t\right\},\text{for}\;\text{all}\;a_{1}\in U.$$

In this case, the hesitancy margin is denoted by $\tau \left(a_{1}\right)=1-\left(\mu_{A_{t}}\left(a_{1}\right)+\nu_{A_{t}}\left(a_{1}\right)\right)$.

**Definition 8** [65]. Let *A*_*t*_ be a t−IFS of universe U. The (*ρ*, *η*)–cut set of t−IFS is a subset of the universe U, which is defined as:

$${Ĉ}_{\left(\rho ,\eta \right)}\left(A_{t}\right)=\left\{a_{1}\;\in U:\;\mu_{A_{t}}\left(a_{1}\right)\geq \rho \;and\;\nu_{A_{t}}\left(a_{1}\right)\leq \eta \right\},$$

where 0 ≤ *ρ* ≤ 1 and 0 ≤ *η* ≤ 1 such that 0 ≤ *ρ* + *η* ≤ 1.

**Definition 9** [64]. The support set $A_{t}^{*}$ of t−IFS *A*_*t*_ of the universe U is defined as:

$$A_{t}^{*}=\left\{a_{1}\;\in U:\;\mu_{A_{t}}\left(a_{1}\right)>0\;and\;\nu_{A_{t}}\left(a_{1}\right)<1\right\}.$$

**Definition 10** [63]. A t−IFS *A*_*t*_ is called t−IFSG of group G, if it admits the following conditions:

1. $\mu_{A_{t}}\left(a_{1}b_{1}\right)\geq \text{min}\left\{\mu_{A_{t}}\left(a_{1}\right),\;\mu_{A_{t}}\left(b_{1}\right)\right\}$,
2. $\nu_{A_{t}}\left(a_{1}b_{1}\right)\leq \text{max}\left\{\nu_{A_{t}}\left(a_{1}\right),\;\nu_{A_{t}}\left(b_{1}\right)\right\}$,
3. $\mu_{A_{t}}\left(a_{1}^{-1}\right)=\mu_{A_{t}}\left(a_{1}\right)$,
4. $\mu_{A_{t}}\left(a_{1}^{-1}\right)=\mu_{A_{t}}\left(a_{1}\right),\;\forall \;a_{1},\;b_{1}\;\in G$.

**Definition 11** [65]. Let *A*_*t*_ be a t−IFSG of group G. The subgroup ${Ĉ}_{\left(\rho ,\eta \right)}\left(A_{t}\right)\;\rho ,\;\eta \;\in \left[0,\;1\right]$ with 0 ≤ *ρ* + *η* ≤ 1 is called a level subgroup of *A*_*t*_.

## 3. Representation of t-intuitionistic fuzzy number and triangular t-intuitionistic fuzzy number

In this section, we define the concepts of t-intuitionistic fuzzy numbers(t-IFℕs) and triangular t-intuitionistic fuzzy numbers(t-IFℕs) and highlight the understanding of these notions by presenting their respective examples and graphs.

**Definition 12.** A t−IFS *A*_*t*_ of U is said to be a t-intuitionistic fuzzy normal set if it admits the following conditions: for any $z_{1}\in U$ such that:

$$\mu_{A_{t}}\left(z_{1}\right)=t\;\text{and}\;\nu_{A_{t}}\left(z_{1}\right)=1\text{-}t.$$

**Definition 13.** A t-IFS *A*_*t*_ defined on a line ℝ is called a t-intuitionistic fuzzy number (t-IFℕ), if *A*_*t*_ satisfies the following conditions:

1. *A*_*t*_ is a t-intuitionistic fuzzy normal set.
2. Each (*ρ*, *η*)–cut set of *A*_*t*_ is a closed interval.
3. The support set of *A*_*t*_ is bounded.

Interestingly, the parameter t is assigned a value of 1, and the t-IFℕ transforms into the classical IFℕ.

**Remark 1.** Every t-IFℕ A_*t*_ is expressible in the form of the following membership and non-membership functions:

$$\mu_{A_{t}}\left(z_{1}\right)=\left\{\begin{array}{c} \begin{array}{ccc} 0 & if & z_{1}\in \left(-\infty ,m_{1}-\alpha_{1}\right]\\ f_{A_{t}}\left(z_{1}\right) & if & \;z_{1}\in (m_{1}-\alpha_{1},m_{1})\\ t & if & \;z_{1}=m_{1} \end{array}\\ \begin{array}{ccc} g_{A_{t}}\left(z_{1}\right) & if & z_{1}\in (m_{1},m_{1}+\beta_{1})\\ 0 & if & z_{1}\in \left[m_{1}+\beta_{1},\infty \right) \end{array} \end{array}\right.$$

and

$$\nu_{A_{t}}\left(z_{1}\right)=\left\{\begin{array}{c} \begin{array}{ccc} 1 & if & z_{1}\in \left(-\infty ,m_{1}-\alpha_{1}^{\prime }\right]\\ h_{A_{t}}\left(z_{1}\right) & if & \;z_{1}\;\in (m_{1}-\alpha_{1}^{\prime },m_{1})\\ 1-t & if & \;z_{1}=m_{1} \end{array}\\ \begin{array}{ccc} k_{A_{t}}\left(z_{1}\right) & if & z_{1}\in \left(m_{1},m_{1}+\beta_{1}^{\prime }\right)\\ 1 & if & z_{1}\in \left[m_{1}+\beta_{1}^{\prime },\infty \right) \end{array} \end{array}\right.$$

where $f_{A_{t}}$ and $g_{A_{t}}$ are strictly increasing and decreasing functions in [*m*_1_ − *α*_1_, *m*_1_] and [*m*_1_, *m*_1_ + *β*_1_] respectively, whereas $k_{A_{t}}$ and $h_{A_{t}}$ are strictly increasing and decreasing functions in $\left[m_{1},m_{1}+\beta_{1}^{\prime }\right]$ and $\left[m_{1}-\alpha_{1}^{\prime },m_{1}\right]$ respectively. Moreover, *m*_1_ denotes the mean value of *A*_*t*_. Additionally, the values *α*_1_ and *β*_1_ are the left and right legs of $\mu_{A_{t}}$, respectively, whereas $\alpha_{1}^{\prime }$ and $\beta_{1}^{\prime }$ are the left and right legs of $\nu_{A_{t}}$ respectively.

The t-IFℕ A_*t*_ is symbolized in the following way: $A_{t-IFN}=\left[m_{1},\alpha_{1},\beta_{1};\alpha_{1}^{\prime },\beta_{1}^{\prime }\right]$.

**Definition 14.** A t-IFℕ is LR-type t-IFℕ such that the membership and non-membership function may be defined as follows:

$$\mu_{A_{t}}\left(z_{1}\right)=\left\{\begin{array}{c} \begin{array}{ccc} 0 & if & z_{1}\in \left(-\infty ,m_{1}-\alpha_{1}\right]\\ min\left\{L\left(\frac{m_{1}-z_{1}}{\alpha_{1}}\right),t\right\} & if & z_{1}\in (\;m_{1}-\alpha_{1},m_{1}),\;\alpha_{1}>0\\ min\left\{1,t\right\} & if & z_{1}=m_{1} \end{array}\\ \begin{array}{ccc} max\left\{R\left(\frac{z_{1}-m_{1}}{\beta_{1}}\right),t\right\} & if & z_{1}\in (\;m_{1},m_{1}+\beta_{1}),\;\beta_{1}>0\\ 0 & if & z_{1}\in \left[m_{1}+\beta_{1},\infty \right) \end{array} \end{array}\right.,$$

and

$$\nu_{A_{t}}\left(z_{1}\right)=\left\{\begin{array}{c} \begin{array}{ccc} 1 & if & z_{1}\in \left(-\infty ,m_{1}-\alpha_{1}^{\prime }\;\right]\\ max\left\{1-L\left(\frac{m_{1}-z_{1}}{\alpha_{1}^{\prime }\;}\right),1-t\right\} & if & z_{1}\;\in \;\text{(}m_{1}-\alpha_{1}^{\prime },\;m_{1}),\;\alpha_{1}^{\prime }\;>0\\ max\left\{0,1-t\right\} & if & z_{1}=m_{1} \end{array}\\ \begin{array}{ccc} max\left\{1-R\left(\frac{z_{1}-m_{1}}{\beta_{1}^{\prime }\;}\right),1-t\right\} & if & z_{1}\in \left(m_{1},m_{1}+\beta_{1}^{\prime }\right),\beta_{1}^{\prime }>0\\ 1 & if & z_{1}\in \left[m_{1}+\beta_{1}^{\prime },\infty \right) \end{array} \end{array}\right..$$

Note that *L*(1) = *R*(1) = 0 and *L*(0) = *R*(0) = 1.

It’s interesting to note that when the parameter t is set to 1, the LR-type t-IFℕ converts into the traditional LR-type IFℕ.

**Example 1.** Consider IFℕ *A* on ℝ is defined as follows:

$$\mu_{A}\left(z_{1}\right)=\left\{\begin{array}{c} \begin{array}{ccc} 0 & if & z_{1}\leq -1\\ 1-\left|z_{1}\right| & if & {-1<z}_{1}<0\\ 1 & if & z_{1}=0 \end{array}\\ \begin{array}{ccc} 1-\left|z_{1}\right| & if & {0<z}_{1}<1\\ 0 & if & \;z_{1}\geq 1 \end{array} \end{array}\right.$$

and

$$\nu_{A}\left(z_{1}\right)=\left\{\begin{array}{c} \begin{array}{ccc} 1 & if & z_{1}\leq -2\\ 0.5\left|z_{1}\right| & if & {-2<z}_{1}<0\\ 0 & if & z_{1}=0 \end{array}\\ \begin{array}{ccc} 0.5\left|z_{1}\right| & if & \;{0<z}_{1}<2\\ 1 & if & \;z_{1}\geq 2 \end{array} \end{array}\right..$$

**Fig 1:**
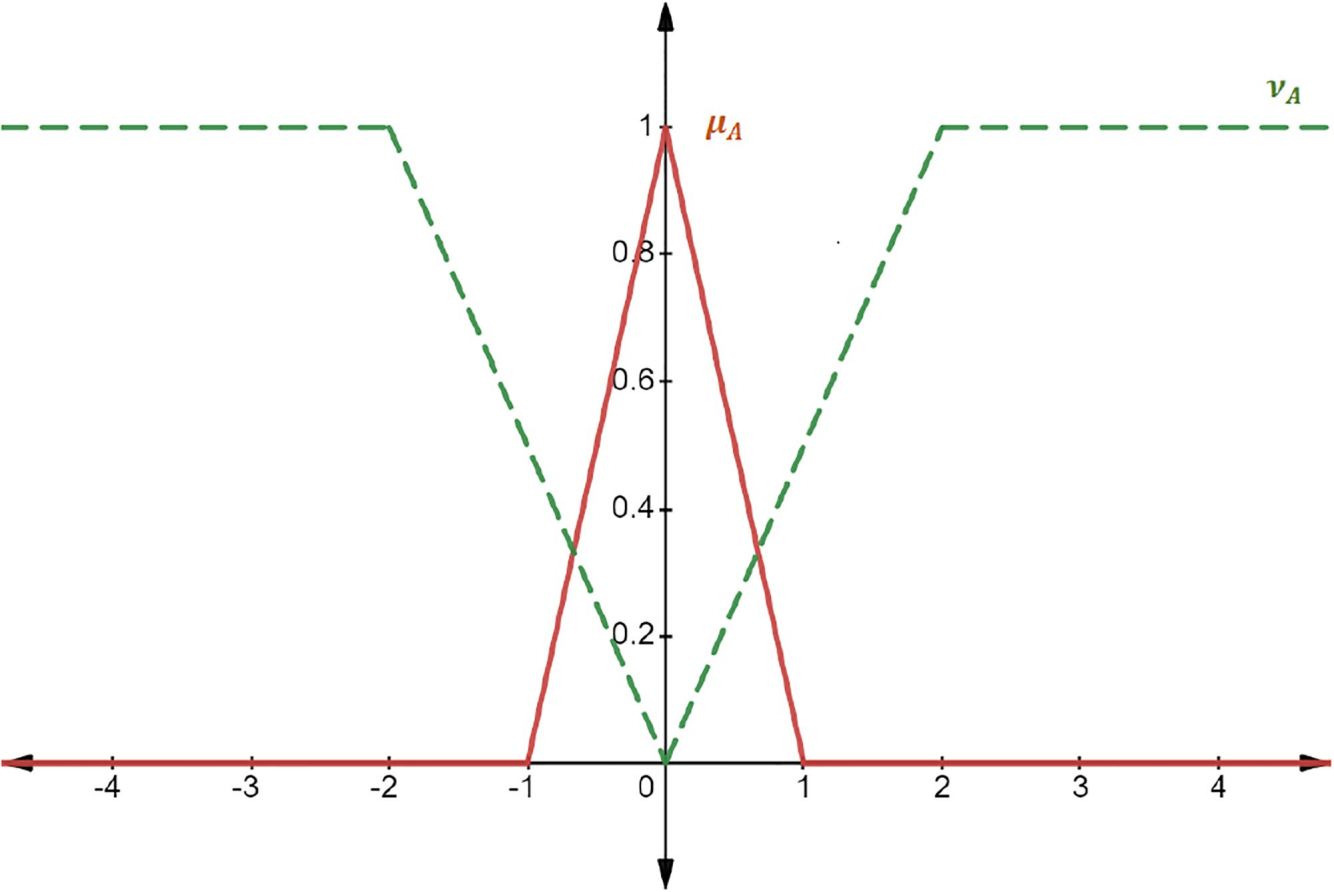
Intuitionistic fuzzy number $A_{IFN}=\left(0,-1,1;-2,2\right)$.

The following Fig 1 shows the graphical representation of IFℕ.

In view of Definition (13), the t-IFℕ corresponds to the value *t* = 0.7 is given by:

$$\mu_{A_{t}}\left(z_{1}\right)=\left\{\begin{array}{c} \begin{array}{ccc} 0 & if & z_{1}\leq -1\\ min\left\{1-\left|z_{1}\right|,0.7\right\} & if & {-1<z}_{1}<0\\ 0.7 & if & z_{1}=0 \end{array}\\ \begin{array}{ccc} min\left\{1-\left|z_{1}\right|,0.7\right\} & if & {0<z}_{1}<1\\ 0 & if & \;z_{1}\geq 1 \end{array} \end{array}\right.$$

and

$$\nu_{A_{t}}\left(z_{1}\right)=\left\{\begin{array}{c} \begin{array}{ccc} 1 & if & z_{1}\leq -2\\ max\left\{0.5\left|z_{1}\right|,0.3\right\} & if & {-2<z}_{1}\\ 0.3 & if & z_{1}=0 \end{array}<0\\ \begin{array}{ccc} max\left\{0.5\left|z_{1}\right|,0.3\right\} & if & {0<z}_{1}<2\\ 1 & if & \;z_{1}\geq 2 \end{array} \end{array}\right..$$

**Fig 2:**
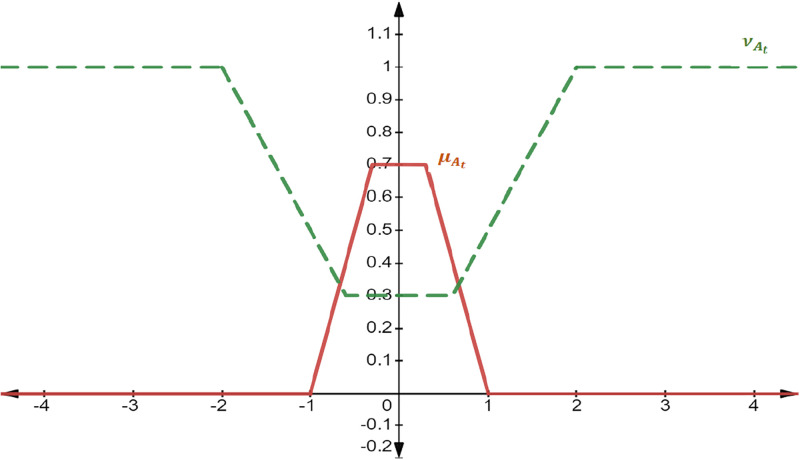
Graphical representation of $0.7-IFN\;A_{0.7-IFN}=\left[0,-1,1;-2,2\right]$.

The following Fig 2 illustrates the graphical representation of 0.7-IFℕ.

**Definition 15.** A t-IFS defined on a line ℝ is said to be triangular t−IFℕ, if it possesses the following membership and non-membership functions as follows:

$$\mu_{A_{t}}\left(z_{1}\right)=\left\{\begin{array}{c} \begin{array}{ccc} 0 & if & z_{1}\in \left(-\infty ,m_{1}-\alpha_{1}\right]\\ min\left\{t\left(\frac{z_{1}-(m_{1}-\alpha_{1})}{\alpha_{1}}\right),t\right\} & if & z_{1}\in \;\text{(}\;m_{1}-\alpha_{1},m_{1}),\;\alpha_{1}>0\\ min\left\{1,t\right\} & if & z_{1}=m_{1} \end{array}\\ \begin{array}{ccc} min\left\{t\left(\frac{(m_{1}+\beta_{1})-z_{1}}{\beta_{1}}\right),t\right\} & if & z_{1}\in (\;m_{1},m_{1}+\beta_{1}),\;\beta_{1}>0\\ 0 & if & z_{1}\in \left[m_{1}+\beta_{1},\infty \right) \end{array} \end{array}\right.,$$

and

$$\nu_{A_{t}}\left(z_{1}\right)=\left\{\begin{array}{c} \begin{array}{ccc} 1 & if & z_{1}\in \left(-\infty ,m_{1}-\alpha_{1}^{\prime }\;\right]\\ max\left\{1+t\left(\frac{(m_{1}-\alpha_{1}^{\prime })-z_{1}}{\alpha_{1}^{\prime }\;}\right),1-t\right\} & if & z_{1}\;\in (m_{1}-\alpha_{1}^{\prime },\;m_{1}),\;\alpha_{1}^{\prime }\;>0\\ max\left\{0,1-t\right\} & if & z_{1}=m_{1} \end{array}\\ \begin{array}{ccc} max\left\{1+t\left(\frac{z_{1}-(m_{1}+\beta_{1}^{\prime })}{\beta_{1}^{\prime }\;}\right),1-t\right\} & if & z_{1}\in \left(m_{1},m_{1}+\beta_{1}^{\prime }\right),\beta_{1}^{\prime }>0\\ 1 & if & z_{1}\in \left[m_{1}+\beta_{1}^{\prime },\infty \right) \end{array} \end{array}\right.,$$

where $\alpha_{1}^{\prime }\leq \alpha_{1}\leq m_{1}\leq \beta_{1}\leq \beta_{1}^{\prime }$. Moreover, *m*_1_ denotes the mean value of *A*_*t*_. The values *α*_1_ and *β*_1_ are the left and right legs of $\mu_{A_{t}}$, respectively, whereas $\alpha_{1}^{\prime }$ and $\beta_{1}^{\prime }$ are the left and right legs of $\nu_{A_{t}}$ respectively. The triangular t-IFℕ *A*_*t*_ is symbolized in the following way:

$$A_{Tr.\;t-IFN}=<m_{1},\alpha_{1},\beta_{1};\alpha_{1}^{\prime },\beta_{1}^{\prime }>.$$

It is important to note that when the parameter t is set to 1, the triangular t-IFℕ converts into the traditional triangular IFℕ.

**Definition 16.** Let *A*_*t*_ be a triangular t-IFℕ. The (*ρ*, *η*)–cut interval of *A*_*t*_ is defined as:

Tρ,ηAt=m1-α1+ρα1t,m1+β1-ρβ1t;-∞,m1-α1′+α1′t1-η⋃m1+β1′-β1′t1-η,∞:0≤ρ,η≤1,0≤ρ+η≤1.

**Remark 2.** For any triangular t-IFℕ $A_{Tr.\;t-IFN}=<m_{1},\alpha_{1},\beta_{1};\alpha_{1}^{\prime },\beta_{1}^{\prime }>,$ then $\alpha_{1}^{\prime }>\alpha_{1},\beta_{1}^{\prime }>\beta_{1}$.

**Example 2.** Consider IFℕ *A* on ℝ is defined as follows:

$$\mu_{A}\left(z_{1}\right)=\left\{\begin{array}{c} \begin{array}{ccc} 0 & if & z_{1}\leq 6\\ \frac{z_{1}-6}{2} & if & {6<z}_{1}<8\\ 1 & if & z_{1}=8 \end{array}\\ \begin{array}{ccc} \frac{10-z_{1}}{2} & if & {8<z}_{1}<10\\ 0 & if & \;z_{1}\geq 10 \end{array} \end{array}\right.$$

and

$$\nu_{A}\left(z_{1}\right)=\left\{\begin{array}{c} \begin{array}{ccc} 1 & if & z_{1}\leq 5\\ \frac{8-z_{1}}{3} & if & {5<z}_{1}<8\\ 0 & if & z_{1}=8 \end{array}\\ \begin{array}{ccc} \frac{z_{1}-8}{4} & if & {8<z}_{1}<12\\ 1 & if & \;z_{1}\geq 12 \end{array} \end{array}\right..$$

**Fig 3:**
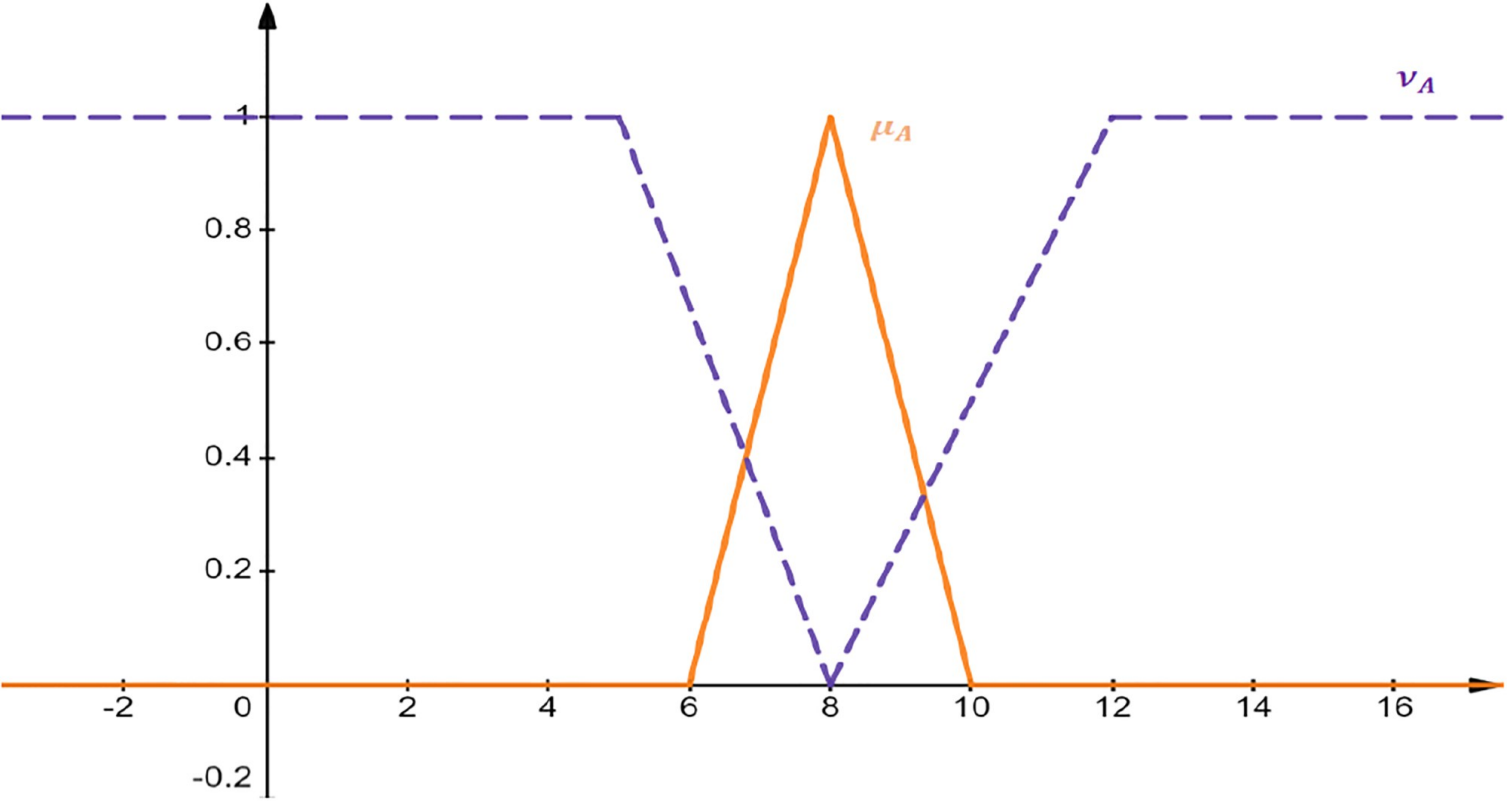
Triangular IFℕ $A_{IFN}=\left\{8,6,10;5,12\right\}$.

The graphical representation of triangular IFℕ is depicted in Fig 3.

In view of Definition (15), the triangular 0.6-IFℕ that is associated with the value *t* = 0.6 can be expressed as follows:

$$\mu_{A_{t}}\left(z_{1}\right)=\left\{\begin{array}{c} \begin{array}{ccc} 0 & if & z_{1}\leq 6\\ min\left\{(0.6)\left(\frac{z_{1}-6}{2}\right),0.6\right\} & if & {6<z}_{1}<8\\ 0.6 & if & z_{1}=8 \end{array}\\ \begin{array}{ccc} min\left\{(0.6)\left(\frac{10-z_{1}}{2}\right),0.6\right\} & if & {8<z}_{1}<10\\ 0 & if & \;z_{1}\geq 10 \end{array} \end{array}\right.$$

and

$$\nu_{A_{t}}\left(z_{1}\right)=\left\{\begin{array}{c} \begin{array}{ccc} 1 & if & z_{1}\leq 5\\ max\left\{1+(0.6)\left(\frac{{5-z}_{1}}{3}\right),0.4\right\} & if & {5<z}_{1}<8\\ 0.4 & if & z_{1}=8 \end{array}\\ \begin{array}{ccc} max\left\{1+(0.6)\left(\frac{z_{1}-12}{4}\right),0.4\right\} & if & {8<z}_{1}<12\\ 1 & if & \;z_{1}\geq 12 \end{array} \end{array}\right..$$

**Fig 4:**
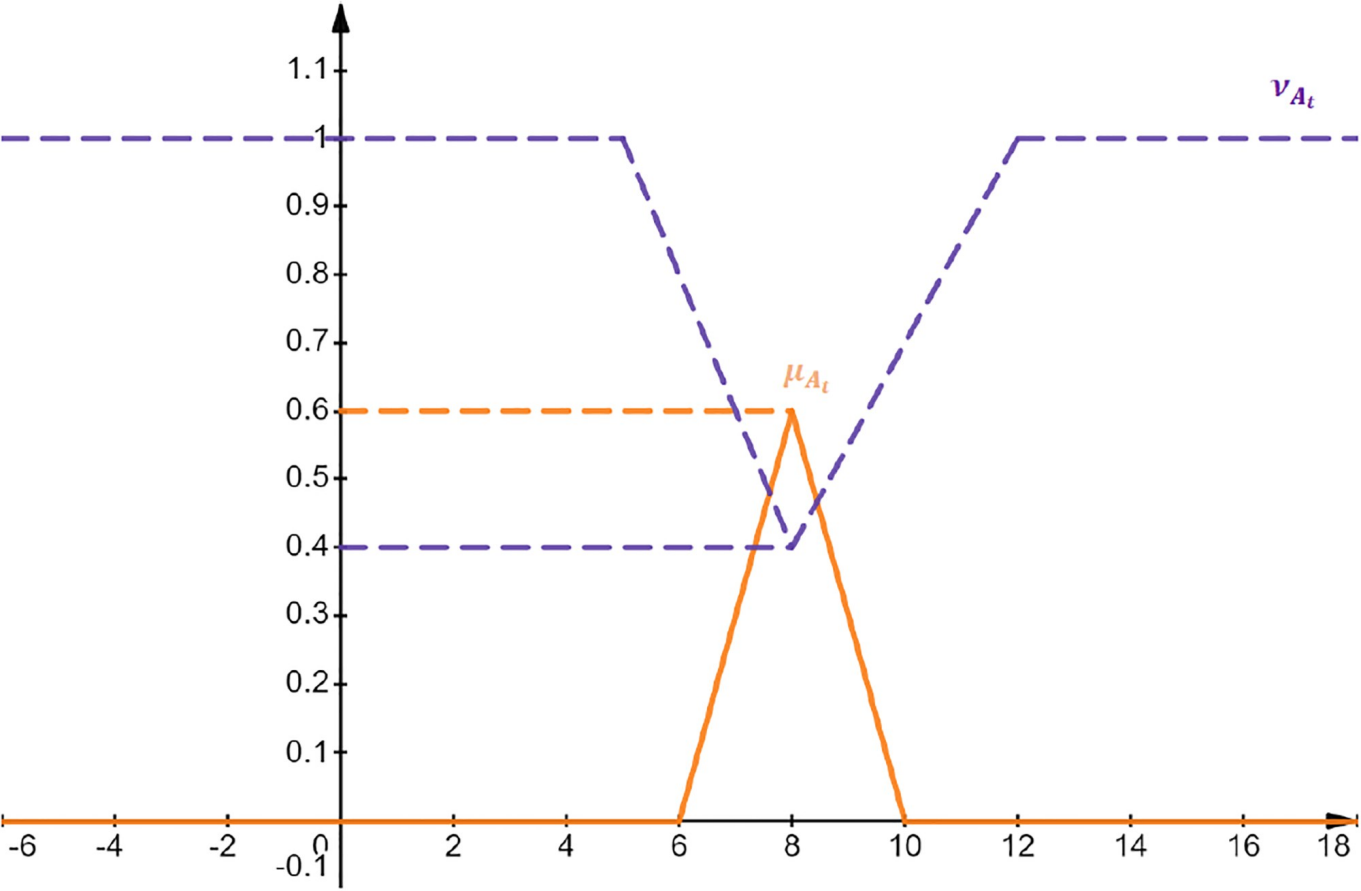
Graphical representation of triangular 0.6-IFℕ $A_{Tr.\;0.6-IFN}=<8,6,10;5,12>$.

The graphical representation of triangular 0.6-IFℕ is given in Fig 4:

**Remark 3.** For any two triangular t-IFℕ $A_{t}=\langle m_{1},\alpha_{1},\beta_{1};\alpha_{1}^{\prime },\beta_{1}^{\prime }\rangle$ and $B_{t}=\langle n_{1},\gamma_{1},\delta_{1};\gamma_{1}^{\prime },\delta_{1}^{\prime }\rangle$, then

1. The addition and subtraction of any two triangular t-IFℕ *A*_*t*_ and *B*_*t*_ is triangular t-IFℕ and is calculated in the following way:
  1. $A_{t}+B_{t}=\langle m_{1}+n_{1},\alpha_{1}+\gamma_{1},\beta_{1}+\delta_{1};\alpha_{1}^{\prime }+\gamma_{1}^{\prime },\beta_{1}^{\prime }+\delta_{1}^{\prime }\rangle$.
  2. $A_{t}-B_{t}=\langle m_{1}-n_{1},\alpha_{1}-\gamma_{1},\beta_{1}-\delta_{1};\alpha_{1}^{\prime }-\gamma_{1}^{\prime },\beta_{1}^{\prime }-\delta_{1}^{\prime }\rangle$.
2. For any scalar *λ*, the scalar multiplication of *λ* and *A*_*t*_ is also triangular t-IFℕ and is determined as follows:
  1. ${\lambda A}_{t}=\langle \lambda m_{1},\lambda \alpha_{1},\lambda \beta_{1};{\lambda \alpha }_{1}^{\prime },\lambda \beta_{1}^{\prime }\rangle$ for *λ* > 0.
  2. ${\lambda A}_{t}=\langle \lambda m_{1},\lambda \beta_{1},\lambda \alpha_{1};\lambda \beta_{1}^{\prime },\lambda \alpha_{1}^{\prime }\rangle$ for *λ* < 0.
3. The exponent of a triangular t-IFℕ is obtained as follows:
$$A_{t}^{p}=\langle m_{1}^{p},pm_{1}^{p-1}\alpha_{1},pm_{1}^{p-1}\beta_{1};pm_{1}^{p-1}\alpha_{1}^{\prime },pm_{1}^{p-1}\beta_{1}^{\prime }\rangle ,$$
  where *p* is a positive integer.

## 4. Application of t-intuitionistic fuzzy subgroup to cryptography

In this section, we present an algorithm that utilizes the concept of a t-IFSG for the RSA cryptosystem.

**Algorithm** t-Intuitionistic Fuzzy RSA Algorithm

**Generation of Keys**:

1: Input any two random distinct prime numbers $p_{1}$ and $q_{1}$.

2: Calculate a modulus $n_{1}=p_{1}q_{1}\;$for both public and private keys.

3: Compute the Euler totient function: $\varphi \left(n_{1}\right)=\left(p_{1}-1\right)\left(q_{1}\;-1\right)$.

4: Choose an encryption exponent $e_{1}$ such that: $1<e_{1}<\varphi \left(n_{1}\right)$ and $gcd\left(e_{1},\;\varphi \left(n_{1}\right)\right)=1$.

5: Compute the decryption exponent d, which satisfies the following congruence: $d_{1}e_{1}\equiv 1\left(mod\varphi \left(n_{1}\right)\right).$

6: The receiver sends the public key $\left(e_{1},n_{1}\right)$ to the sender and retains the private key $\left(d_{1},n_{1}\right).$

7: **Output** public and private keys.

**Encryption Phase**:

8: **Input** public key and $n_{1}.$

9: The sender received the recipient’s public key $\left(e_{1},n_{1}\right).$

10: Represents the experimental message s into an integer or plain text S in view of Table 1.

11: Compute a t-IFSG corresponding to the set S.

12: Determine the (*ρ*, *η*)–level subgroup of t-IFSG.

13: Compute the t-IFℕ of the level subgroup for the set of integers.

14: Obtain the triangular t-IFℕ from t-IFℕ.

15: Compute ciphertext by using the RSA encryption formula $CT\equiv S^{e_{1}}\left(modn_{1}\right)$.

16: The sender sends the ciphertext in the form of a triangular t-IFℕ.

17: **Output** ciphertext.

**Decryption Phase**:

18: **Input** private key, ciphertext and $n_{1}$.

19: The message is retrieved by employing the RSA decryption formula $PT\equiv {CT}^{d_{1}}\left(modn_{1}\right)$, using a private key $\left(d_{1},n_{1}\right)$ and triangular t-IFℕ exponentiation operation is employed.

20: The message is in triangular t-IFℕ form and is verified by applying the Definition of congruence and subtraction of triangular t-IFℕ.

21: Obtain t-IFℕ from triangular t-IFℕ.

22: Obtain the set of integers.

23: Obtain the plain text from Table 1.

24: **Output** plaintext.

**Table 1:** Experimental message into the integer.

s	A	B	C	D	E	F	G	H	I	J	K	L	M
S	01	02	03	04	05	06	07	08	09	10	11	12	13
s	N	O	P	Q	R	S	T	U	V	W	X	Y	Z
S	14	15	16	17	18	19	20	21	22	23	24	25	16

**Fig 5:**
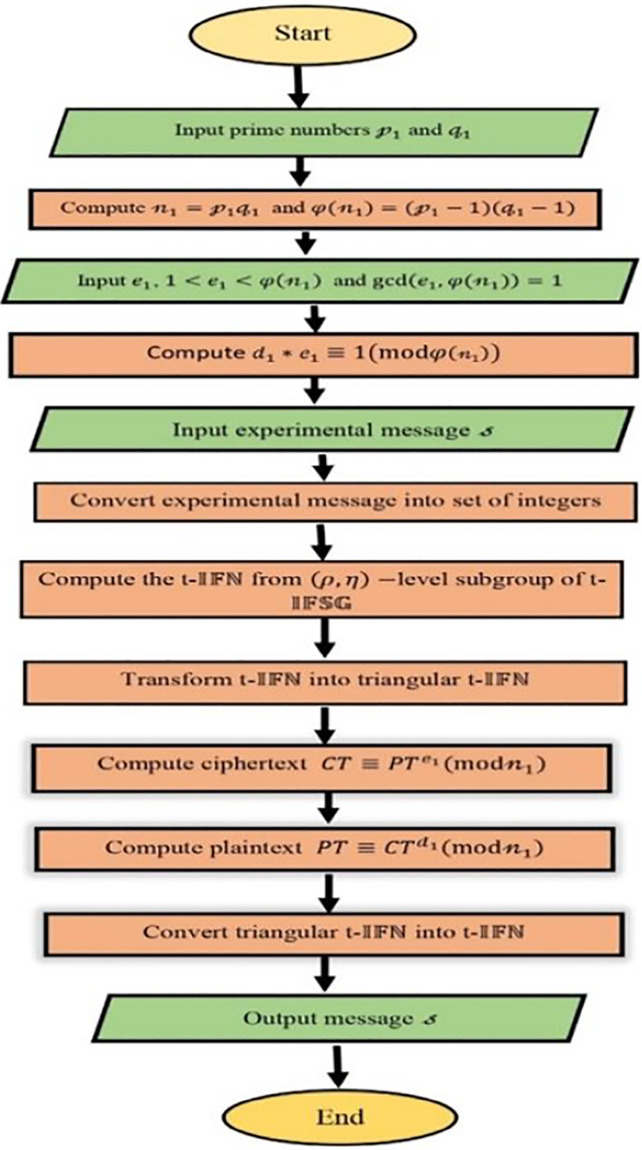
Process of t-intuitionistic Fuzzy RSA Cryptosystem.

The flowchart shown in Fig 5 clearly explains the RSA algorithm in the framework of t-IFSG.

### 4.1 Numerical example of t-intuitionistic fuzzy RSA algorithm

This section is devoted to presenting a numerical example that illustrates the application of t-IFSG to encrypt and decrypt a message using the t-intuitionistic fuzzy RSA module. The process of a t-intuitionistic fuzzy RSA Cryptosystem consists of three steps: generating keys, encryption, and decryption. The subsequent example depicts the mechanism of this method, using an experimental message, "Rose."

- **Generate public and private keys:**
  Choose two distinct prime numbers, such as $p_{1}=5$ and $q_{1}=11.$
  Compute $n_{1}=p_{1}q_{1}$ giving: $n_{1}=55$.
  Compute Euler totient functions such as: *φ*(55) = 40.
  Choose any number $e_{1}=7$ *such as* $1<e_{1}<40$ and $gcd\left(e_{1},\;40\right)=1$.
  Choose *d*_1_ = 23 that satisfies: 7*d*_1_ ≡ 1(*mod*40).
- **Encrypting the plaintext:**
  The public key is (7,55) and the private key is (23,55).
  The experimental message is taken as "Rose."
  Convert the experimental message into the set of integers: {18,15,19,05}.
  Obtain a 0.7-IFSG corresponding to the set S as follows:
$$\mu_{A_{0.7}}\left(z_{1}\right)=\left\{\begin{array}{cc} 0.7 & if\;z_{1}\in <0>\\ 0.5 & if\;z_{1}\in <2>-<0>\;\\ 0.4 & if\;z_{1}\in Z_{26}-<2> \end{array}\right.$$
  and
$$\nu_{A_{0.7}}\left(z_{1}\right)=\left\{\begin{array}{cc} 0.3 & if\;z_{1}\in <0>\\ 0.4 & if\;z_{1}\in <2>-<0>\\ 0.5 & if\;z_{1}\in Z_{26}-<2> \end{array}\right..$$
  Compute the (0.4,0.5)–level subgroup of the above 0.7-IFSG as follows: ${Ĉ}_{\left(0.4,0.5\right)}\left(A_{t}\right)=S$.
  In view of Definition (14), 0.7-IFℕ of ${Ĉ}_{\left(0.4,0.5\right)}\left(A_{t}\right)$ for the experimental message "Rose" is given by:
$$\left[1,1,18;1,19\right]\left[1,1,15;1,16\right]\left[1,1,19;1,20\right]\left[1,1,5;1,6\right].$$
  Transform the above 0.7-IFℕ into Triangular 0.7-IFℕ:
<1,0,17;1,19><1,0,14;1,16><1,0,18;1,20><1,0,4;1,6>.
  Encrypt the Triangular 0.7-IFℕ using a public key as follows:
<1,0,9;7,23><1,0,43;7,2><1,0,16;7,30><1,0,28;7,42>.
- **Decrypting the ciphertext:**
  The receiver receives the ciphertext in Triangular 0.7-IFℕ:
<1,0,9;7,23><1,0,43;7,2><1,0,16;7,30><1,0,28;7,42>.
  Decrypt the ciphertext using a private key as follows:
<1,0,42;51,34><1,0,54;51,46><1,0,38;51,30><1,0,39;51,31>.
  Verify the ciphertext with plaintext *modulo* 55.
  The original message is in Triangular 0.7-IFℕ as follows:
<1,0,17;1,19><1,0,14;1,16><1,0,18;1,20><1,0,4;1,6>.
  Convert Triangular 0.7-IFℕ into 0.7-IFℕ:
$$\left[1,1,18;1,19\right]\left[1,1,15;1,16\right]\left[1,1,19;1,20\right]\left[1,1,5;1,6\right].$$
  Transform 0.7-IFℕ into the set of integers:{18,15,19,05}.
  The plain text is "Rose".

Encrypting and decrypting processes will benefit user communication by making it difficult for attackers to break the message.

### 5. C++ Program to implement the t-intuitionistic fuzzy RSA algorithm

**Fig 6:**
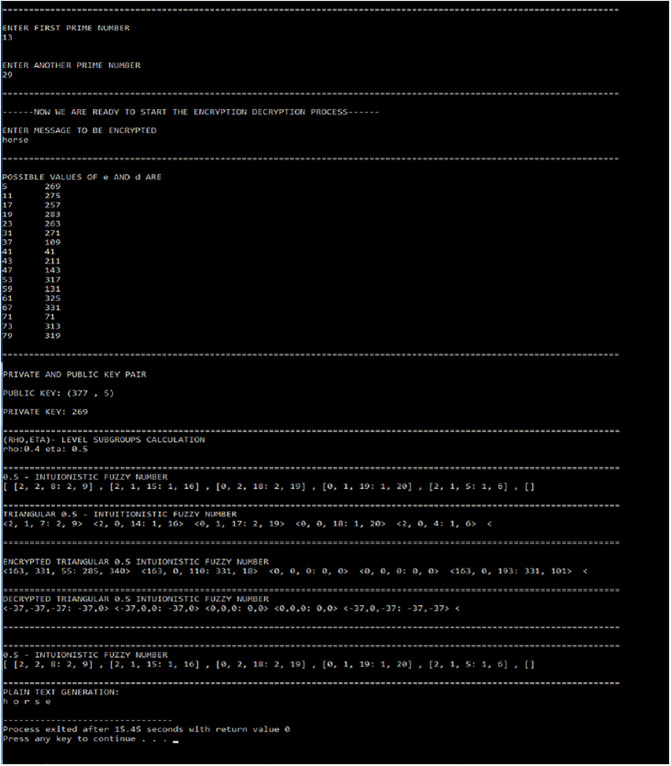
Execution of t-intuitionistic fuzzy RSA algorithm.

In this section, we implement the t-IF-RSA Cryptosystems in computer language. The t-IF-RSA algorithm is computationally viewed in Fig 6.

## 6. Comparative analysis and limitations of the current study

This section presents a comparative analysis of the proposed and existing RSA techniques and discusses the proposed mechanism’s limitations.

One can establish a comparative analysis of the proposed algorithm with the existing RSA algorithm and a fuzzy version of the RSA algorithm. This comparison shows that the newly proposed algorithm to secure a message is better than the current algorithms. Table 2 shows the comparative comparison between the t-IF-RSA algorithm and existing algorithms.

**Table 2:** Comparative comparison between RSA algorithm, fuzzy RSA algorithm, t-IF-RSA algorithm.

Algorithms	Key differences
**RSA Algorithm**	After 8 seconds, the output is displayed. The hacker hacks the algorithm by knowing the value of a private key. If the hacker decrypts the decrypted text, he can quickly determine the encrypted text using the Extended Euclidean algorithm. The hacker can easily crack the plaintext.
**RSA Algorithm in the Framework of Fuzzy Set** [67]	The program is not coded. The algorithm does not start with a parameter. It means it’s hackable. If the hacker hacks the algorithm, he can’t easily encrypt text. The hacker can’t easily hack the plaintext because the plaintext depends on the Fℕ and triangular Fℕ. The fuzzy RSA algorithm employs fuzzy sets to represent uncertainty, where each element is assigned a degree of membership. The adaptability of the algorithm to changing and unpredictable environments is credited to its basis in fuzzy logic. The introduction of additional complexity has the potential to improve security. However, implementing formal security assurances may pose a challenge. Human proficiency can influence the RSA algorithm that uses fuzzy sets when parameterizing the fuzzy sets. Incorporating fuzzy logic in this method can increase its complexity, potentially impacting its computational efficiency. The capabilities for fine-tuning are comparatively lesser in this approach than in the t-intuitionistic fuzzy RSA scheme. This framework describes the suitability of a particular strategy for situations that exhibit moderate uncertainty and necessitate flexibility.
**RSA Algorithm in the Framework of t-Intuitionistic Fuzzy Subgroup**	For at least 10 seconds, the output is displayed. The hacker can’t crack the algorithm despite knowing the private key because it depends upon the value of the parameters *t*, *ρ* and *η* where *t*, *ρ*, *η* ∈ [0,1]. If the hacker hacks the decrypted text, he can’t encrypt the text because the proposed algorithm is more secure than the existing algorithms. The hacker can’t crack the plaintext easily because the plaintext depends on the t-IFℕ and triangular t-IFℕ. The t-IF-RSA scheme incorporates the parameter "t," allowing t-norm and t-conorm operators to manage uncertainty effectively. Introducing the parameter "t" in the algorithm increases its ability to respond to ambiguity, thereby improving adaptability. The tool provides custom settings via the parameter "t," allowing adaptive security levels. Formal security analysis may provide difficulties owing to its increased complexity. Extensive protocol synthesis and expert knowledge have enhanced and optimized the algorithmic adjustment feature, enabling it through the parameter "t." The added complexity introduced by the "t" parameter necessitates a comprehensive assessment of computational efficiency. By allowing modifications to the security levels, the parameter "t" facilitates precise system adjustments and enables modifications to security levels. This approach aims to adjust and modify to meet the specific security needs of a wide range of applications.

The following are the limitations of the proposed techniques:

- In certain situations, the t-intuitionistic fuzzy approach may not be appropriate if the sum of the degree of membership and non-membership of the elements is greater than 1. To address this challenge, we suggest exploring alternative approaches in more flexible contexts, such as Pythagorean fuzzy, fermentation fuzzy, picture fuzzy, and spherical fuzzy environments.
- We have added extra security measures to improve the current cryptosystem, specifically to the difficulty of guessing the secret key. We have used t-intuitionistic fuzzy graph theory to strengthen the secret key, which has proven highly effective in overcoming these limitations.

## 7. Conclusions and future work

Cryptography is the technique used to protect the security of an individual’s or an organization’s data or private communications that must be kept secret or confidential. It is one of the methods to secure a message with a key unique to the sender or receiver. In t-IFSG, The RSA method resolves concerns and hesitations in subgroup structural cryptography. This improves group-based cryptographic processes, encompassing ambiguous connections and hesitations about complex subgroup memberships. This cryptographic approach can describe the degrees of belonging, non-belonging, and hesitation flexibly and securely, which obeys more than binary logic. In this research paper, we have introduced the notions of t-IFℕ and triangular t-IFℕ. A practical and advanced method for cryptographic security, i.e., the t-IF-RSA scheme based on the RSA scheme, has been introduced. In this cryptographic scheme, it has been observed that the strategy of managing the fluctuation of the uncertainty level becomes more rapid. For the proposed cryptographic scheme, making the scheme with the parameter ‘t’ seems much more felicitous because the security countermeasure could be customized according to their specific requirements or a threat scenario. Furthermore, we have presented a comparative study of this newly developed mechanism with the existing RSA algorithm and have highlighted its significance.

Future directions for enhancing encryption in t-intuitionistic fuzzy environments include improved vital management, performance optimization, security analysis, integration with emerging technologies, real-world implementation, and standardization efforts. The present ideology shall be extended to the t-intuitionistic fuzzy graph theory, focusing on its practical application across various domains, including cryptography. The exploration of this theory seeks to achieve a heightened level of security.
